# Inhibition during task switching is affected by the number of competing tasks

**DOI:** 10.3758/s13421-023-01456-w

**Published:** 2023-09-12

**Authors:** Juliane Scheil, Thomas Kleinsorge

**Affiliations:** Leibniz Research Centre for Working Environment and Human Factors, Ardeystraße 67, D-44139 Dortmund, Germany

**Keywords:** *N* – 2 repetition costs, Inhibition, Task switching

## Abstract

Inhibition during task switching is assumed to be indexed by *n* – 2 repetition costs—that is, performance costs when the task in the current trial equals the task in trial *n* – 2 (sequences of type ABA) compared with two consecutive switches to another task each (sequences CBA). The present study examined effects of a short-term reduction of the number of candidate tasks on these costs. For this purpose, a variant of the task switching paradigm was used in which in half of the trials, a precue that preceded the task cue allowed for a short-term reduction of the number of candidate tasks. In Experiment 1, one out of three tasks could be excluded. In Experiment 2, one or two out of four tasks could be excluded. Experiment 3 served as control condition using the standard cueing paradigm. Significant *n* – 2 repetition costs were present with three candidate tasks. In contrast, no costs were visible when the number of candidate tasks was reduced to two. This result is interpreted in terms of a task selection mechanism based on antagonistic constraints among task representations, which operates on a rather superficial level when switching among only two tasks, thereby reducing the need for inhibition.

## Introduction

The ability to deal with a permanently changing environment is central for human flexible control of behavior. In cognitive psychology, the task switching paradigm is commonly used as a tool to investigate characteristics of cognitive processes underlying this flexibility. When switching among different tasks, we are confronted with the need to activate relevant information on the one hand and at the same time prevent interference from currently irrelevant information. While the former may be achieved by activating some kind of task-set, the latter may be accomplished by inhibitory processes (cf. Kiesel et al., 2010; Koch et al., 2018, for reviews). In task switching, inhibition can be assessed by using the so-called *n* – 2 repetition costs (Mayr & Keele, 2000): when switching among (at least) three tasks A, B, and C, reaction times and sometimes error rates are higher when the task in the current trial equals the task in trial *n* – 2 (sequences of type ABA) compared with two consecutive switches to another task each (sequences CBA). This effect is commonly explained by the occurrence of task inhibition after each switch trial that persists for some time and, therefore, has to be overcome when the current task was inhibited in trial *n* – 2.

During the past years, cognitive research has tried to elucidate characteristics of the inhibitory processes underlying *n* – 2 repetition costs in more detail. One important aspect concerns the interplay of inhibition and (some kind of) activation processes during task switching. If *n* – 2 repetition costs reflect inhibitory processes in task switching, then this means that the absence of *n* – 2 repetition costs is not equal to an absence of inhibition, because this would cause an *n* – 2 repetition benefit (Grange et al., 2013). Instead, zero *n* – 2 repetition costs reflect a reduced level of inhibition.

Regarding the activation of relevant task information, previous work by Kleinsorge and Scheil (2015, 2017) investigated how a short-term change of the task environment affects cognitive task representations. Specifically, they used a variant of the task switching paradigm in which, in addition to the regular task cue that indicates the relevant task, a precue was presented at the beginning of each trial. In half of the trials, this precue was informative in terms of excluding two of the four tasks as the relevant one in the upcoming trial, thereby reducing the number of candidate tasks to two. With these informative precues, switch costs were significantly reduced as compared with uninformative precues that did not reduce the number of candidate tasks, and this reduction was mainly due to faster reactions in switch trials, while task repetitions were almost unaffected. These results were interpreted as an indication that the activation of cognitive representations during task switching depend on the interrelations between tasks. When switching among two tasks, any evidence for one of the tasks carries at the same time evidence against the other one. In terms of the formal model of task switching put forward by Meiran et al. (2008), if activation of one task component is *x*, the corresponding activation of the other task is 1 – *x*, implying that activation levels of both tasks sum up to 1. Such antagonistic constraints allow for a discrimination between tasks on the basis of any feature that differentiates between the tasks. As suggested by Kleinsorge and Apitzsch (2012), this peculiarity of two-task environments may also account for the observation that precues that discriminate between tasks on the level of perceptual features speed up task switching among two tasks more than mere foreknowledge of the next task, which is not the case when switching among four tasks. This is because with more than two tasks, a task switch indicated on the perceptual level of precues does not translate directly into evidence for one specific task because there is more than one competing task.

In order to observe *n* – 2 repetition costs, at least three tasks are necessary. In line with the above considerations, it can be assumed that participants must determine the relevant task on the basis of more complex interrelations among tasks than simple antagonistic constraints. This suggests that the methodological requirement of employing more than two tasks in order to be able to measure *n* – 2 repetition costs may impose certain requirements on the process of task selection that have been neglected in previous research. Thus, the central tenet of the present article is that the need for establishing these more complex interrelations among competing tasks relates to the representational foundation of the need for task-set inhibition and, therefore, affects the level of observed *n* – 2 repetition costs.

The notion of antagonistic constraints, as well as its corollary of different task-selection mechanisms with two versus more tasks is also implicit in other theories of task switching that build on the idea of intertask conflict. It is assumed that during task switching, conflicts arise among the different tasks, and that dealing with these conflicts is a key goal of cognitive control processes (cf. Schuch et al., 2019, for a review). In terms of the computational model of Sexton and Cooper (2017), each task is considered as being represented by a task demand unit that has a certain activation level. If two task demand units have activation levels larger than zero, the resulting conflict is detected by conflict monitoring units. Each conflict monitoring unit receives input from two (and only two) of the task demand units. This means that in situations with three tasks, three conflict monitoring units are assumed to be active. As soon as a conflict is detected, these units affect task processing via inhibitory connections to the task demand units in order to reduce activation of the currently irrelevant task(s). The exclusion of a task as the possibly relevant one by the precue might reduce the activation of its task demand unit and, as a consequence, minimize interference ensuing from that task, thereby reducing inhibition released by the conflict monitoring units. In case of a reduction of the number of candidate tasks from three to two, this means that only one rather than three conflict monitoring units may be needed to handle intertask conflict. On a behavioral level, this might lead to reduced *n* – 2 repetition costs.

We are not the first to suggest that *n* – 2 repetition costs are affected by variations of the interrelations among individual tasks. With respect to task dominance, which is a paradigmatic case for an interrelation between tasks, Jost et al. (2017) found larger *n* – 2 repetition costs for a dominant compared with a weaker task. Furthermore, Gade and Koch (2014) observed effects of different cue types on *n* – 2 repetition costs, with nontransparent cues (colored frames) causing higher costs compared with transparent ones (task names). This effect was interpreted in terms of transparent cues leading to distinct (that is, more easily to discriminate) task representations that are less susceptible to interference. Similarly, Arbuthnott (2005) could show that cueing tasks by unique spatial locations results in *n* – 2 benefits instead of costs, leading her to suggest that “spatial location serves to increase discrimination, and thus relative activation, of the current task set, reducing the need for lateral inhibition of other options prior to response selection” (p. 1041). Moreover, it has been shown that *n* – 2 repetition costs decrease with practice (Grange & Juvina, 2015; Scheil, 2016), which can be explained by cue–target associations becoming stronger with increasing practice, leading to faster retrieval. Summarizing these results in a broader context, it can be stated that *n* – 2 repetition costs are sensitive to the difficulty of task selection and task set activation, with task discriminability seemingly being a key factor in this respect. As a consequence, the question arises whether *n* – 2 repetition costs might be affected by the possibility to form simple antagonistic constraints on a short-term basis, which would allow task discrimination to proceed in a much more elementary manner as compared with interrelations among three candidate tasks that require more complex forms of discrimination.

For this purpose, we adapted the paradigm used by Kleinsorge and Scheil (2015) to a variant designed to measure *n* – 2 repetition costs in two experiments. In Experiment 1, participants switched among three tasks, and the number of candidate tasks was reduced to two in half of the trials. In Experiment 2, participants switched among four tasks in total. In 33% of all trials, no task could be excluded. In 33%, the number of candidate tasks was reduced to three. In 33%, the number was reduced to two. We hypothesize that a short-term reduction of the number of candidate tasks reduces the complexity of the task environment by allowing the formation of simple antagonistic constraints among the two remaining tasks. This, in turn, should reduce the need for inhibition and lead to smaller *n* – 2 repetition costs. To anticipate results, significant *n* – 2 repetition costs were found with three candidate tasks, while with two remaining candidate tasks, no costs were visible. In the condition with four candidate tasks that was implemented in Experiment 2, an *n* – 2 repetition benefit was found. To investigate whether this effect was due to characteristics of the special variant of the paradigm, we used the standard cueing variant of the task switching paradigm in Experiment 3 and varied the number of tasks between blocks. Experiment 3 therefore served as a control experiment.

## Experiment 1

### Method

#### Participants

Thirty subjects (seven male) with a mean age of 24.0 years (range: 19–29 years) participated. All had normal or corrected-to-normal vision. For power estimation, we used the interaction of Task Transition and Precue that was found in Experiment 1 of Kleinsorge and Scheil (2015), as the design of this experiment offers the highest similarity with the present one. With an effect size of ${\upeta}_{\textrm{p}}^2$ = .66 and using MorePower 6.0 (Campbell & Thompson, 2012), this yielded a power estimation of .99 for the present sample size.

#### Stimuli, tasks, and apparatus

Stimuli consisted of two different shapes (x and +) presented in yellow or blue and with a size of either 3 cm × 3 cm or 6 cm × 6 cm. Task cues consisted of a dark-grey diamond, square, or triangle surrounding the position of the imperative stimulus with a size of about 7 cm × 7 cm. Participants switched among three perceptual decision tasks in which they had to judge the stimuli regarding their size (large vs. small, indicated by the diamond), color (yellow or blue, indicated by the square), or their shape (x or +, indicated by the triangle).

**Fig. 1:**
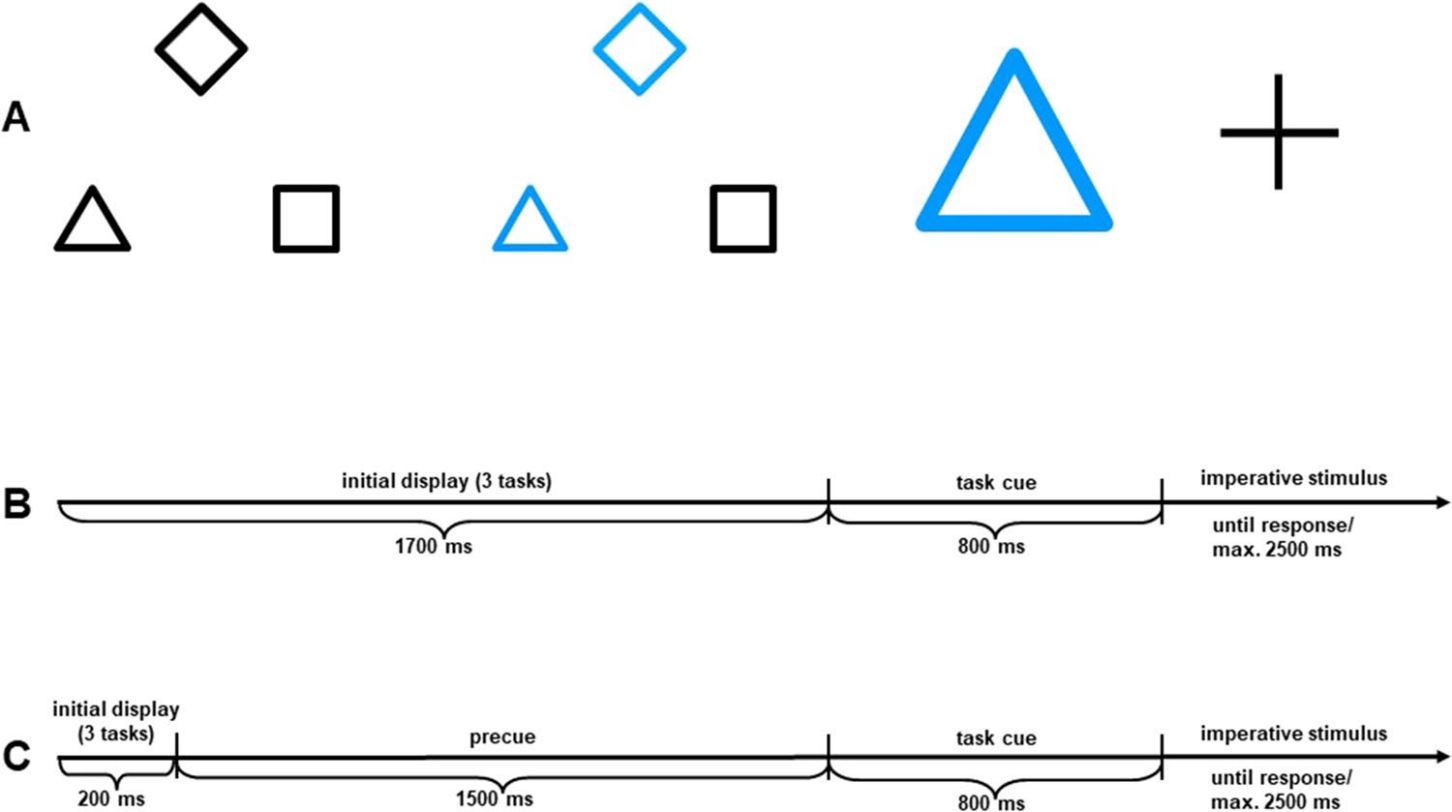
Experiment 1: Illustration of the stimuli (**A**); schematic illustration of the procedure of a single trial with informative precue (**B**); schematic illustration of the procedure of a single trial with noninformative precue (**C**). Note: Precues that were colored grey in the experiment are depicted in black to allow for grey-scale copy

Precues consisted of the three task cues presented simultaneously with a size of 3 cm × 3 cm each. They surrounded the center of the screen in form of a triangle (cf. Fig. 1). The position of the precues was held constant for each participant to prevent orienting responses but was balanced across participants. Initially, all three precues were colored in grey. In the noninformative condition, they stayed grey during their presentation time. In the informative condition, two of the three precues turned blue, meaning that one of the tasks whose precues changed color would be relevant next. In this condition, one precue of the two currently irrelevant tasks was chosen at random to be presented together with the relevant one. Whether the precue was informative or not was varied in a trial-wise fashion and counterbalanced across the three tasks.

All tasks occurred with equal frequency. Task repetitions occurred in 33% of trials. The inclusion of task repetitions in the present experiment was necessary because otherwise, only two tasks could be relevant in the next trial, making the precue meaningless. Direct stimulus repetitions were not allowed. Stimuli were presented centrally on a light-grey background. Viewing distance was not controlled but approximated 60 cm. Participants pressed the “y” key of a German QWERTZ keyboard for small, blue, and x-shaped stimuli and the “-“ key for big, yellow, and +-shaped stimuli.

#### Design and procedure

After giving informed consent, participants were provided with on-screen instructions in which the tasks and the meaning of the task cues were explained. Instructions emphasized speed as well as accuracy. The experiment was run in a single session that took about 70 minutes. It started with a practice block of 80 trials. In this block, all tasks were practiced separately for 16 trials each. After that, 32 mixed trials without task repetitions followed. The test session consisted of 12 experimental blocks of 96 trials each.

The procedure of single trials is depicted in Fig. 1. Each trial began with the presentation of the initial display in which all precues were colored grey. In the uninformative condition, this initial display was presented for 1,700 ms. In the informative condition, the initial display was presented for 200 ms. After that, two of the precues turned blue and remained on the screen for 1,500 ms. Then the task cue was presented for 600 ms in both conditions. After that, the cue disappeared and the imperative stimulus was presented for 2,500 ms or until the participant’s response. In case of an error, error feedback was presented for additional 1,000 ms; in case of RTs slower than the RT deadline of 2,500 ms, RT feedback was presented for additional 1,000 ms. After the response or error feedback, the next initial display was presented immediately.

### Results

#### Main analysis

The practice block was not analyzed. Furthermore, the first three trials of each block were excluded, as were sequences involving an error in trials *n* – 2 or *n* – 1. Furthermore, sequences involving at least one repetition were excluded (i.e., sequences of type AAA, AAB, and ABB, leading to a data loss of 53.7%). From RT data, errors in the current trial were also excluded. A mean number of 29 trials per cell (*SD* = 3) was left for analyses.

As *n* – 2 repetition costs are calculated over a series of three consecutive trials, not only effects of the precue in the current trial but also effects of the precue in trials *n* – 1 and *n* – 2 are included in the analyses. These factors are labelled lag1_Precue and lag2_Precue, respectively. In a first step, RT and ER data were analyzed separately. However, as a trend towards a speed–accuracy trade-off was visible, we decided to combine both measures. As a consequence, linear integrated speed–accuracy scores (LISAS; Vandierendonck, 2018) were calculated and analyzed using a repeated-measures analysis of variance (ANOVA) with the within-subjects factors task sequence (CBA vs. ABA), precue (informative vs. noninformative), lag1_Precue, and lag2_Precue.

**Fig. 2:**
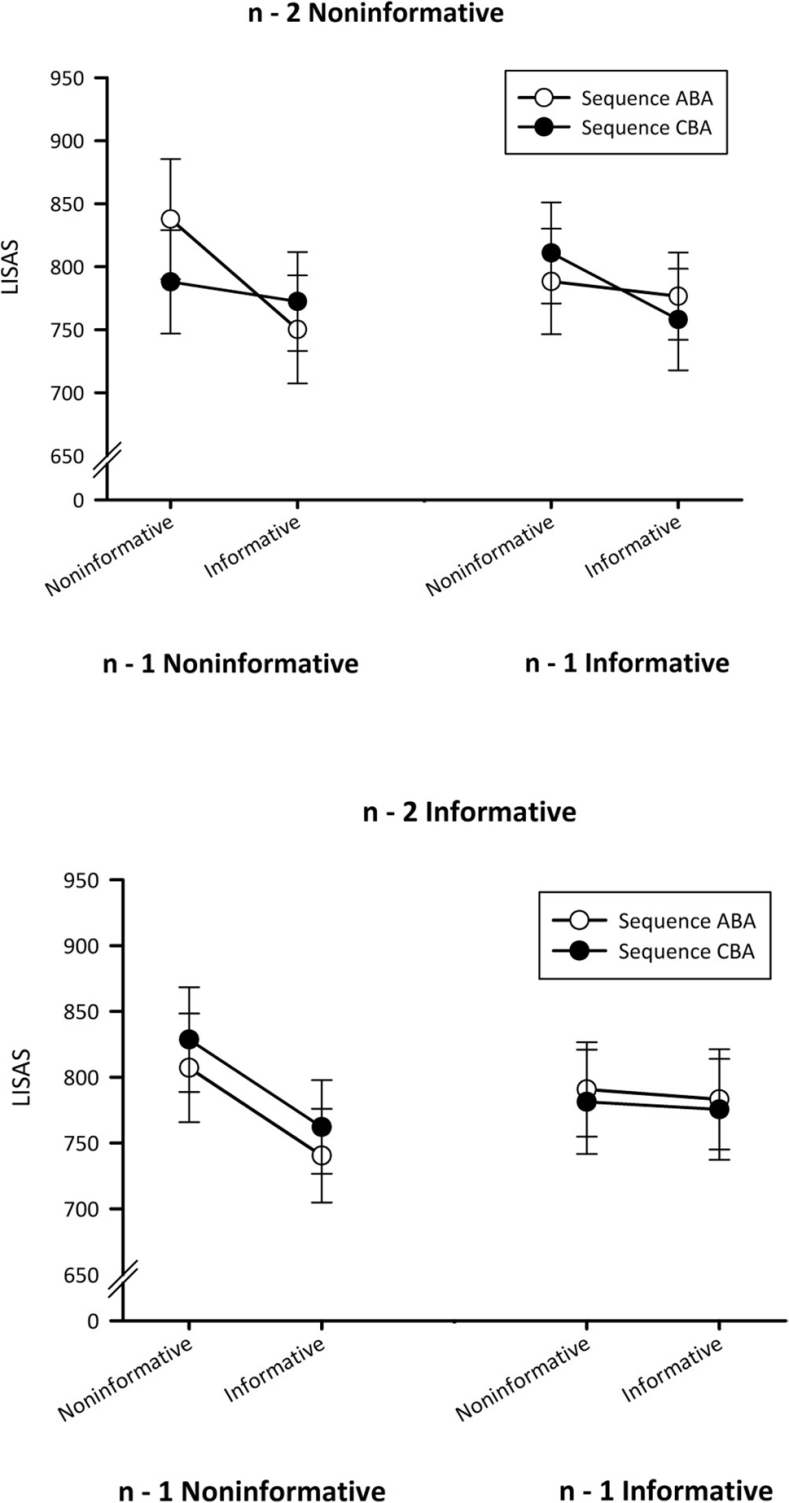
Experiment 1: Mean LISAS as a function of task sequence, precue, lag_precue, and lag2_precue. Error bars represent *SEM*

A significant main effect of precue occurred, *F*(1, 29) = 22.35, *p* < .001, ${\upeta}_{\textrm{p}}^{{}^2}$ = .44. Informative precues reduced mean LISAs from 804 to 765. Precue and lag1_Precue interacted, *F*(1, 29) = 10.16, *p* < .01, ${\upeta}_{\textrm{p}}^{{}^2}$ = .26. Informative precues reduced LISAs from 815 to 765 when preceded by a noninformative precue (*p* < .011, Duncan corrected) but only from 793 to 773 when preceded by an informative one (*p* < .05). Most importantly, the four-way interaction of all factors was significant, *F*(1, 29) = 5.80, *p* < .05, ${\upeta}_{\textrm{p}}^{{}^2}$ = .17. Significant (*p* < .05, Duncan corrected) *n* – 2 repetition costs where only present when all three trials of the sequence involved noninformative precues (cf. Fig. 2). In all other conditions, *n* – 2 repetition costs did not significantly differ from zero (all *p*s > .22). To analyze *n* – 2 repetition costs in the different conditions, we ran Bayes factor (BF) paired *t* tests to compare ABA and CBA sequences in the different conditions. We used JASP (JASP Team, 2023) for all Bayes analyses. The Bayes factor yields an estimate of the likeliness for the alternative hypothesis H1 compared with the null hypothesis H0. In this case BF_10_ refers to the likeliness of the presence of *n* – 2 repetition costs compared with a zero effect. In sequences with three noninformative precues, the contrast of ABA versus CBA sequences yielded a BF_10_ of 9.14, which can be interpreted as substantial evidence for H1 (Jeffreys, 1961). In all other conditions, the BF_10_ was below 1 (between 0.21 and 0.38 for the different conditions), so no *n* – 2 repetition costs were present.

#### Supplementary analyses

##### Identity of excluded tasks

**Fig. 3:**
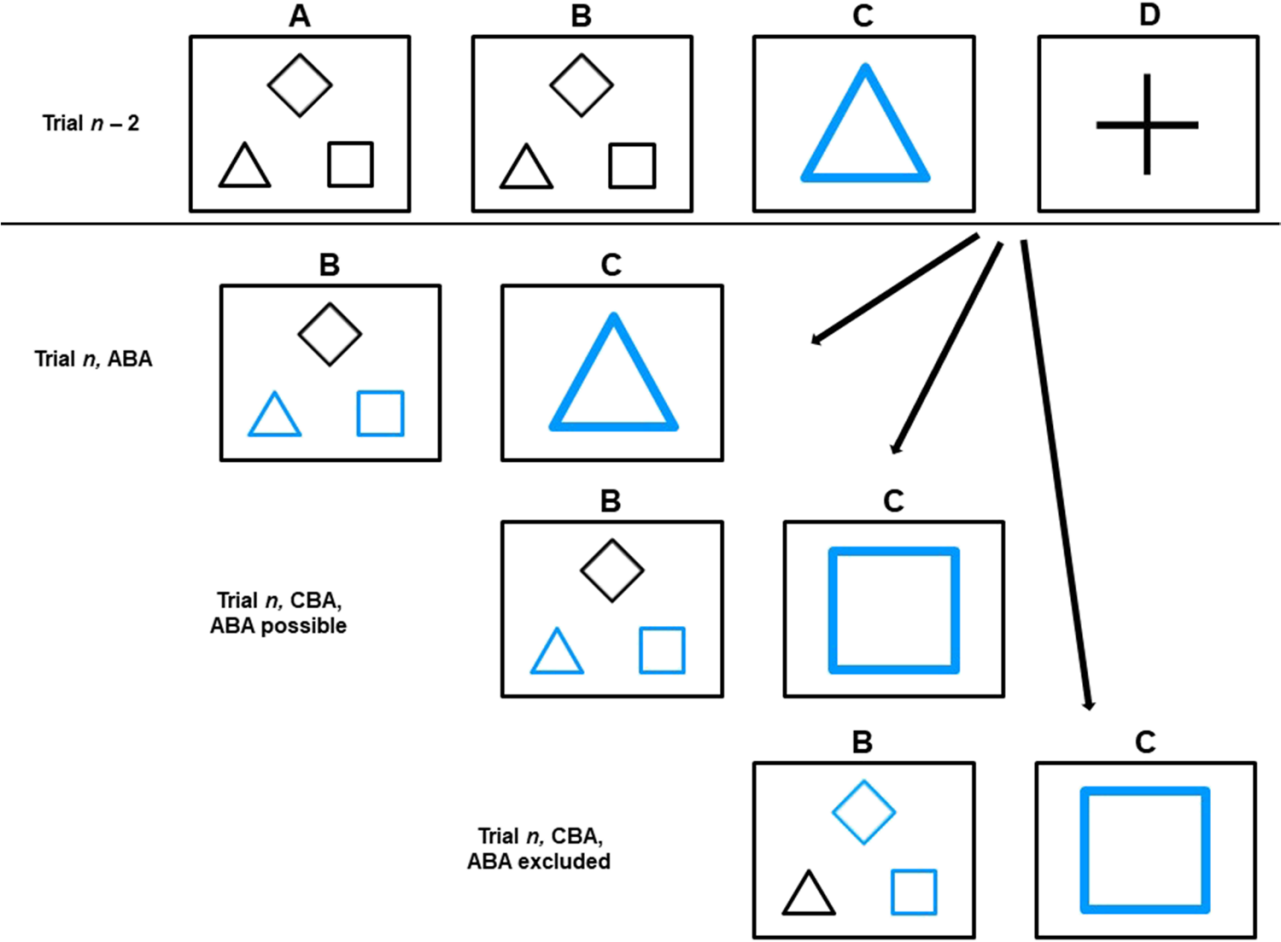
Illustration of the different trial types in case of an informative precue in trial *n*. Upper panel: Display of trial *n* – 2 with noninformative precue (the precue condition of this trial is not of importance): (**A**) initial display (**B**) precue display, noninformative (**C**) task cue (**D**) imperative stimulus. Lower panel: Illustration of (**B**) informative precue displays and (**C**) task cues of trial *n.* Note: Precues that were colored grey in the experiment are depicted in black to allow for grey-scale copy

How *n* – 2 repetition costs are affected by the precue might depend on the identity of the excluded task. Specifically, one may assume that it makes a difference whether in CBA sequences, an informative Precue allows or excludes an ABA sequence. The difference between these trial types is depicted in Fig. 3.

To further investigate this possibility, LISAS of informative precues were analyzed with a repeated-measures ANOVA, with the within-subjects factor task sequence (ABA, CBA with ABA possible, CBA with ABA excluded). However, this analysis yielded no significant effect (*F* = 1.69, *p* = .19 for the main effect of task sequence). On a descriptive level, LISAS were highest for CBA sequences in which an ABA sequence was possible (786), followed by ABA sequences (774) and CBA sequences in which an ABA sequence was ruled out (764).

##### Effects of precues on switch costs

The main result of the studies of Kleinsorge and Scheil (2015, 2017) were reduced switch costs with informative precues. To check whether this finding was replicated in the present study, we analyzed mean LISAS using a repeated-measures ANOVA, with the within-subjects factors task transition (task repetition vs. task switch) and precue (informative vs. noninformative). For data filtering, the first trial of each block was discarded, as were posterror trials. For calculating mean individual reaction times, errors in the current trial were also excluded.

The ANOVA yielded a main effect of task transition, *F*(1, 29) = 69.75, *p* < .001, ${\upeta}_{\textrm{p}}^{{}^2}$ = .71, because LISAS were higher for task switches (797) than for task repetitions (696). Furthermore, the main effect of precue was significant, *F*(1, 29) = 18.10, *p* < .001, ${\upeta}_{\textrm{p}}^{{}^2}$ = .38, because an informative precue reduced mean LISAS from 759 to 735. Most importantly, the interaction of both factors was significant, *F*(1, 29) = 8.13, *p* < .01, ${\upeta}_{\textrm{p}}^{{}^2}$ = .22. Informative precues reduced switch costs from 111 to 90.

##### Effects of stimulus congruency

One requirement for effects of the precue on the formation of task sets is that participants indeed build task sets instead of resorting to other ways to keep the relevant information in memory. One way to verify that task sets are actually present is to look at effects of stimulus congruency. In the present study, three-dimensional stimuli were used that entail relevant information for all three tasks. As a consequence, a stimulus may afford the same response for all three tasks (congruent stimuli). Alternatively, it may afford one possible response for the currently relevant task and another response for the currently irrelevant tasks (incongruent stimuli). In experiments with three tasks, the stimulus may also be congruent with regard to one of the alternative tasks and incongruent with regard to the third task (mixed stimuli). Usually, reaction times and error rates are higher for incongruent than for congruent stimuli, and this difference is larger for task switches than for task repetitions (see Vandierendonck et al., 2010, for a review), which may be explained by task shielding. With changing environmental demands, our cognitive system has to shield itself from currently irrelevant information that may interfere with optimal processing in line with task demands. However, when rapidly switching among tasks, this shielding has to be temporarily relaxed in order to be able to flexibly adjust to the frequent changes (Dreisbach & Wenke, 2011). Therefore, shielding is supposed to be fully exerted in task repetitions but to be reduced in task switches, which makes the cognitive system more vulnerable for the interfering effect of incongruent stimuli. Crucially, task shielding does only occur when task sets are built, whereas no shielding is accomplished when subjects use simple stimulus response rules for performing a task (Dreisbach & Haider, 2009). Thus, an interaction of task transition and stimulus congruency would strongly suggest that participants indeed used task sets in the present experiment.

To investigate effects of stimulus congruency, we analyzed mean LISAS using a repeated-measures ANOVA, with the within-subjects factors task transition (task repetition vs. task switch), stimulus congruency (congruent, mixed, incongruent), and precue (informative vs. noninformative). For data filtering, the first trial of each block was discarded, as were posterror trials. For calculating mean individual reaction times, errors in the current trial were also excluded. Only effects involving stimulus congruency are reported.

The ANOVA yielded a main effect of stimulus congruency, *F*(2, 58) = 86.81, *p* < .001, ${\upeta}_{\textrm{p}}^{{}^2}$ = .75. LISAS were smallest for congruent stimuli (689), intermediate for mixed stimuli (731), and highest for incongruent ones (788). Stimulus congruency and task transition interacted, *F*(2, 58) = 3.24, *p* < .05, ${\upeta}_{\textrm{p}}^{{}^2}$ = .10, because the congruency effect was larger for task switches (LISAS of 731, 781, and 844 for congruent, mixed, and incongruent stimuli, respectively) than for task repetitions (LISAS of 647, 680, and 733 for the three conditions). There was a trend for a three-way interaction of all factors, *F*(2, 58) = 3.03, *p* = .056, that was driven by the fact that switch costs for congruent and incongruent stimuli tended to be smaller with an informative that noninformative precue.

The interaction of task transition and stimulus congruency is in line with previous literature showing task shielding for task repetitions but relaxed shielding for task switches, which results in higher congruency effects in the latter condition (Dreisbach & Haider, 2009). This suggest that participants indeed used task sets in the present experiment.

In an additional step, we had a closer look at stimuli with mixed congruency, meaning that the stimulus presented with the task in trial *n* is congruent with one of the alternative tasks but incongruent with the other one. If task sets are activated (or shielded) to support performance, one could assume that the identity of the task in trial *n* – 1 has an influence on processing the stimulus. More precisely, we assume that performance is worse if the stimulus in trial *n* is incongruent with the task that has been activated in trial *n* – 1. In this condition, lingering activation may hamper the retrieval of the correct response. In contrast, performance is assumed to be better if the stimulus in trial *n* is congruent with the task of trial *n* – 1 and, at the same time, incongruent with the third task, which has been activated less recently. To investigate this, mean individual LISAS were subjected to a paired *t* test with partial stimulus congruency (congruent vs. incongruent with the task in trial *n* – 1). Only task switches with stimuli with mixed congruency were included in this analysis. The effect of partial stimulus congruency was significant, *t*(29) = 1.91, *p* < .05 (one-tailed). LISAS were higher when the stimulus in trial *n* was incongruent with the task in trial *n* – 1 (798) compared with the congruent condition (785). This further corroborates the notion that task sets are continuously activated and inhibited to guide task switching performance.

### Discussion

Using a special variant of the task switching paradigm, we manipulated the number of candidate tasks in a trial-wise fashion. The reduction of candidate tasks from three to two had an effect on *n* – 2 repetition costs: Significant *n* – 2 repetition costs were present in sequences in which no task could be excluded based on the precues. As soon as a reduction was possible in at least one of the trials, *n* – 2 repetition costs vanished. This result is in line with the assumption that a task environment with only two tasks allows for a task set activation on a rather superficial level, therefore reducing the need for inhibition. However, before interpreting the results in more detail, further emphasis should be put on possible effects of the experimental design. The present experiment deviates from paradigms usually used to investigate *n* – 2 repetition costs. Task repetitions were included, which is usually not the case when focusing on *n* – 2 repetition costs. Moreover, conditions with three and two tasks differed with respect to the amount of information participants had to process. With three tasks, no change in the initial display occurred, so participants could just ignore the precues. With two tasks, on the contrary, the display changed and participants had to process the precues, thereby also enhancing working memory load. To check whether these differences affected the data pattern found in Experiment 1, we conducted a new experiment in which participants generally switched among four tasks without task repetitions. Precues reduced the number of candidate tasks from four to three or from four to two. This led to an equal trial procedure for the conditions with three and two tasks. In addition, the cue–target interval (CTI) was varied between blocks in order to check whether the relatively long CTI employed in Experiment 1 (800 ms) led to the absorption of some effects into the CTI. If so, these should become visible in RTs with the shorter CTI of 200 ms employed in this experiment. We expected to replicate the reduction of *n* – 2 repetition costs when the number of candidate tasks is reduced from four to two, whereas costs should be present with a reduction from four to three. For the baseline condition with four tasks, we expect significant *n* – 2 repetition costs to occur. Based on the idea of antagonistic constraint, no specific predictions can be made related to this condition, because no differences of task interrelations with more than two tasks are assumed in the model. Based on the computational model of Sexton and Cooper (2017), *n* – 2 repetition costs may be even higher in the condition with four tasks, because the amount of potential between-task conflict as well as the number of conflict monitoring units is even higher compared with the condition with three tasks.

## Experiment 2

### Method

#### Participants

Thirty subjects (five male) with a mean age of 24.0 years (range: 19–30 years) participated. All had normal or corrected-to-normal vision. As in Experiment 1, our main interest focused on an interaction of task transition and precue. Assuming an effect size of ${\upeta}_{\textrm{p}}^{{}^2}$ = .17, as it was the case for the four-way interaction of Experiment 1, MorePower yielded an estimated power of .86 for this sample size.

##### Stimuli, tasks, and apparatus

Stimuli consisted of two different shapes (x and +) with a size of either 3 cm × 3 cm or 6 cm × 6 cm, presented with either a dashed or a solid line that was either thin (2 pt) or thick (6 pt). Task cues consisted of a dark-grey diamond, square, triangle, or circle surrounding the position of the imperative stimulus with a size of about 7 cm × 7 cm. Participants switched among four perceptual decision tasks in which they had to judge the stimuli regarding their size (large vs. small, indicated by the diamond), shape (x or +, indicated by the triangle), line type (dashed or solid, indicated by the square), or line thickness (thin or thick, indicated by the circle).

Precues consisted of the four task cues presented simultaneously with a size of 3 cm × 3 cm each. They surrounded the center of the screen in form of a diamond. The position of the precues was held constant for each participant to prevent orienting responses but was balanced across participants. In 33% of all trials, no task could be excluded based in the precues. In this condition, all precues were colored grey during their presentation time. In 33% of the trials, two of the four precues turned blue, meaning that one of the tasks whose precues changed color would be relevant next. In this condition, one precue of the two currently irrelevant tasks was chosen at random to be presented together with the relevant one. In 33% of the trials, three of the four precues turned blue, creating a condition that visually excluded one of the tasks. Importantly, it was always the task that has been relevant in trial *n* – 1 (i.e., a task repetition) that was excluded. As task repetitions were not allowed during this experiment, this was therefore a mock exclusion that provided no additional information to the noninformative condition but was comparable to the exclusion of two tasks regarding the display settings. The precue condition was varied in a trial-wise fashion and was counterbalanced across the four tasks. Furthermore, the cue target interval (CTI) was varied in a block-wise fashion. In half of the blocks, the task cue was presented for 200 ms, while the CTI was 600 ms for the other half of the blocks. Blocks with long and short CTI alternated, the length of the first CTI was varied between subjects.

All tasks occurred with equal frequency without task repetitions. Direct stimulus repetitions were not allowed. Stimuli were presented centrally on a light-grey background. Viewing distance was not controlled but approximated 60 cm. Participants pressed the “y” key of a German QWERTZ keyboard for small, x-shaped, dashed, and thin stimuli and the “-“ key for big, +-shaped, solid, and thick stimuli.

##### Design and procedure

This was identical to Experiment 1, except for the following differences.

The session started with a practice block of 96 trials. In this block, all tasks were practiced separately for 16 trials each. After that, 32 mixed trials without task repetitions followed.

Each trial began with the presentation of the precue display for 1,200 ms. Then, the task cue was presented for either 200 ms or 600 ms. After that, the cue disappeared and the imperative stimulus was presented for 2,500 ms or until the participant’s response.

### Results

The practice block was not analyzed. Furthermore, the first three trials of each block were excluded, as were sequences including an error in trials *n* – 2 or *n* – 1. From RT analyses, errors in the current trial were also excluded. A mean number of 18 trials per cell (*SD* = 4) was left for analyses. Mean individual LISAS were analyzed using repeated-measures ANOVAs with the within-subjects factors task sequence (CBA vs. ABA), number of possible tasks (NTasks) in trial *n* (N4 vs. N3 vs. N2), NTasks in trial *n* – 1, NTasks in trial *n* – 2, and CTI (200 ms vs. 600 ms).

The main effect of Task Sequence was not significant, *F* = 1.45, *p* = .23. The main effect of NTasks was also not significant, *F* = 2.26, *p* = .11, as was the main effect of NTasks in *n* – 1, *F* = 0.88, *p* = .42. The main effect of NTasks in *n* – 2 was significant, *F*(2, 58) = 3.23, *p* < .05, ${\upeta}_{\textrm{p}}^{{}^2}$ = .10. Post hoc tests (Duncan corrected) indicated that LISAS were significantly (*p* < .05) higher with four (998) compared with two candidate tasks in trial *n* – 2, (978), while the N3 condition did not differ from the other two (987, *p* = .16 and *p* = .27, respectively). The main effect of CTI was significant, *F*(1, 29) = 289.36, *p* < .001, ${\upeta}_{\textrm{p}}^{{}^2}$ = .91, because LISAS were higher with a short (1,097) than with a long (879) CTI.

Coming to the two-way interactions, the interaction of NTasks and NTasks in trial *n* – 1 was significant, *F* (4, 116) = 4.88, *p* < .01, ${\upeta}_{\textrm{p}}^{{}^2}$ = .14. When no task was excluded in trial *n* – 1, there were no significant differences between the three levels of NTasks in trial *n* (*p*s > .35). The same holds for conditions with two candidate tasks in trial *n* – 1 (ps > 05). With three candidate tasks in trial *n* – 1, all three levels of NTasks in trial *n* differed from each other (*p*s < .05). LISAS were shortest in the condition N2 in trial *n* (x-N3-N2, LISAS of 953), intermediate in the x-N3-N4 condition (983), and highest in the x-N3-N3 condition (1014). To sum up the data pattern of this interaction, the factor NTasks had only an effect in conditions with three candidate tasks in trial *n* – 1. In these conditions, LISAS were shortest with two candidate tasks, intermediate with four tasks, and highest with three tasks. Furthermore, the interaction of NTasks and NTasks in trial *n* – 2 was significant, *F*(4, 116) = 2.92, *p* < .05, ${\upeta}_{\textrm{p}}^{{}^2}$ = .09. When no task was excluded in trial *n* – 2, there were no significant differences between the three levels of NTasks in trial *n* (*p*s > .19). The same holds for conditions with three candidate tasks in trial *n* – 2 (*p*s > .59). With two candidate tasks in trial *n* – 1, LISAS were significantly (*p*s < .05) shorter in the N2-x-N2 condition (969) than in the N2-x-N3 condition (1018) and the N2-x-N4 condition (1007), while the latter two did not differ (*p* = .39). In sum, the factor NTasks had only an effect with two tasks in trial *n* – 2. In these conditions, LISAS were shortest with two candidate tasks than with three or four tasks. Furthermore, the interaction of NTasks in trial *n* – 1 and NTasks in trial *n* – 2 was significant, *F* (4, 116) = 2.90, *p* < .05, ${\upeta}_{\textrm{p}}^{{}^2}$ = .09. When no task was excluded in trial *n* – 2, there were no significant differences between the three levels of NTasks in trial *n* – 1 (*p*s > .63). The same holds for conditions with three candidate tasks in trial *n* – 2 (*p*s > .29). With two candidate tasks in trial *n* – 2, LISAS were significantly faster (*p*s < .05) in the N2-N3-x condition (975) than in the N2-N4-x (1,011) and the N2-N2-x condition (1,007), while the latter two did not differ (*p* = .74). In sum, the factor NTasks in trial *n* – 1 had only an effect with two candidate tasks in trial *n* – 2. In these conditions, LISAS were shorter after three candidate tasks than after two or four tasks.

**Fig. 4:**
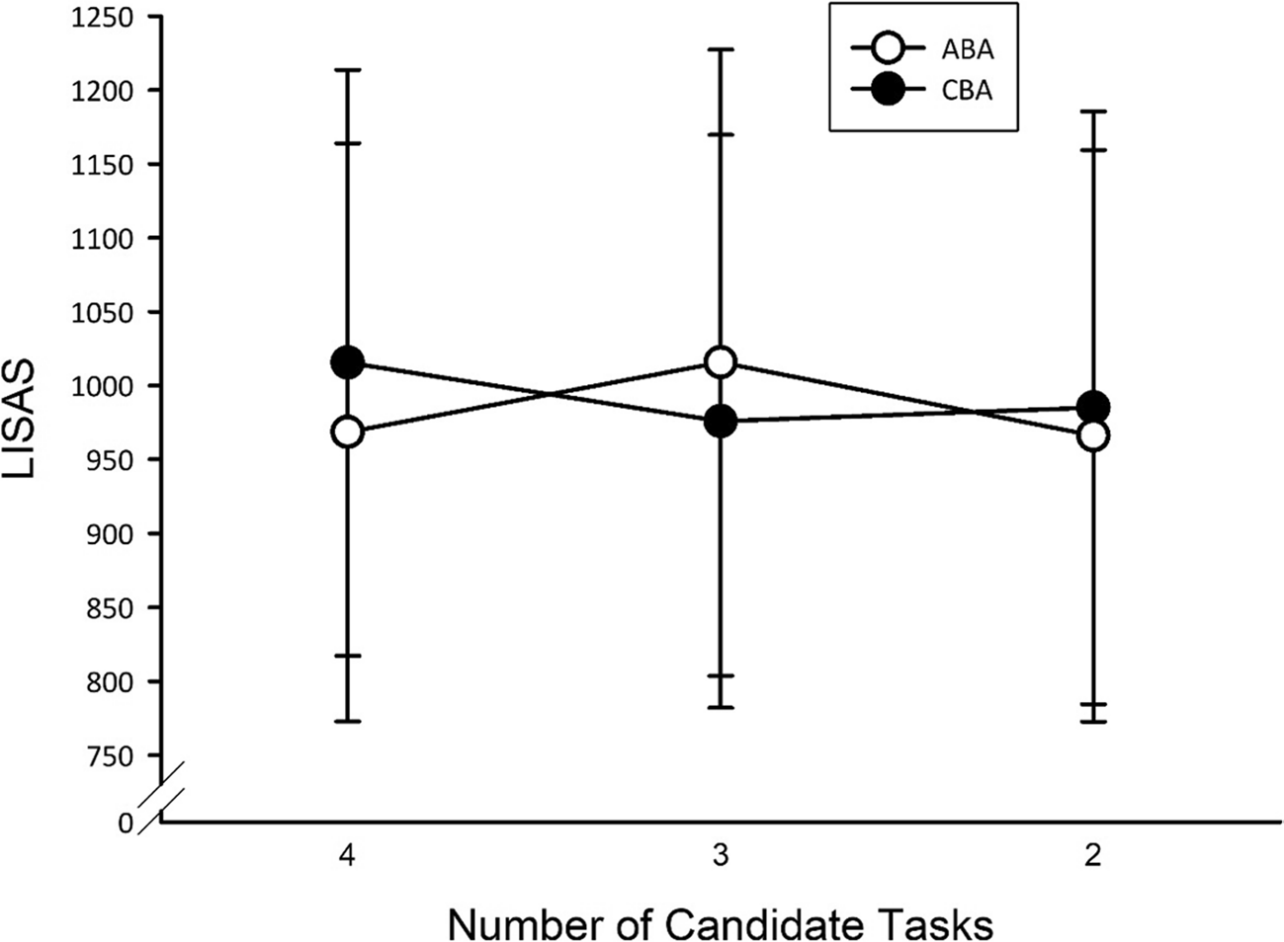
Experiment 2: Mean LISAS as a function of task sequence and number of candidate tasks in trial *n*. Error bars represent *SEM*

Most importantly, the interaction of task sequence and NTasks was significant, *F*(2, 58) = 12.82, *p* < .001, ${\upeta}_{\textrm{p}}^{{}^2}$ = .31. Significant (*p* < .01) *n* – 2 repetition costs were visible in the N3 condition, while they were reduced to zero in the N2 condition (*p* = .17). In terms of Bayes statistics, there was strong evidence for the presence of *n* – 2 repetition costs with three tasks (BF_10_ = 14.13) and anecdotal evidence for an *n* – 2 repetition benefit (i.e., inverted *n* – 2 repetition costs) with two tasks (BF_10_ = 1.08). In N4, a significant *n* – 2 repetition benefit occurred (*p* < .001, BF_10_ = 6.74; cf. Fig. 4). The interactions of task sequence and NTasks in trial *n* – 1 or NTasks in trial *n* – 2 were not significant (*F* = 2.73, *p* = .08, and *F* = 0.19, *p* = .83, respectively).

**Fig. 5:**
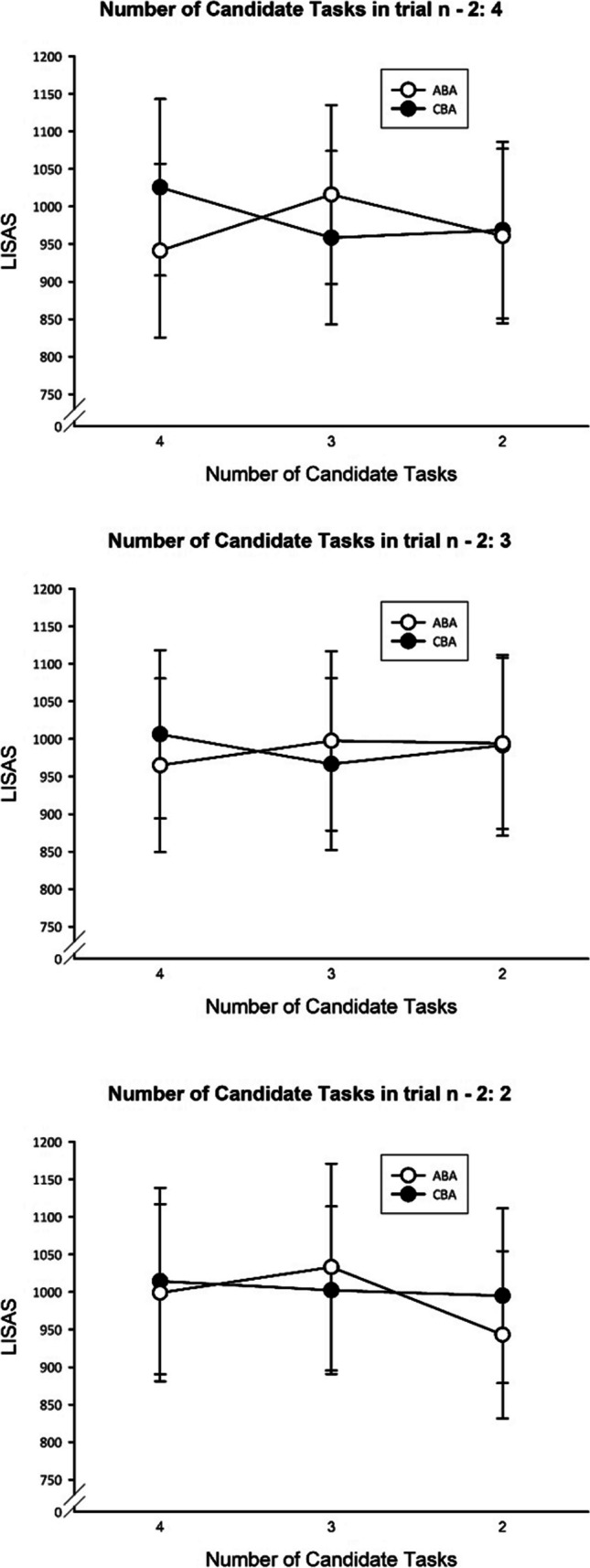
Experiment 2: Mean LISAS as a function of task sequence, number of candidate tasks in trial *n*, and number of candidate tasks in trial *n* – 2. Error bars represent *SEM*

Coming to the three-way interactions, task sequence was modulated by NTasks and NTasks in trial *n* – 2, *F*(4, 116) = 3.91, *p* < .01, ${\upeta}_{\textrm{p}}^{{}^2}$ = .12. As can be seen in Fig. 5, the interaction of NTasks and task sequence was most pronounced when no reduction of candidate tasks was possible in trial *n* – 2. The other two-way interactions were not significant (all *F*s < 2.56, all *p*s > .08).

None of the four-way interactions was significant (all *F*s < 1.27, all *p*s > 26). The five-way interaction of all factors was not significant, *F* = 0.31, *p* = .96.

### Discussion

The effect of main interest, the reduction of *n* – 2 repetition costs with a reduction of the number of candidate tasks to two, could be replicated. Noteworthy, despite its large main effect, none of the interactions with the CTI were significant. This replicates previous findings of no effect of CTI variations on *n* – 2 repetition costs when the CTI is varied in a block-wise fashion (e.g., Mayr, 2002; Schuch & Koch, 2003), or when only effects of the CTI in trial *n* are considered (Gade & Koch, 2008; Scheil & Kleinsorge, 2014).

However, what was not expected was the *n* – 2 repetition benefit in the baseline condition with four tasks. We hypothesized no difference from the N3 condition, especially as the reduction from four to three tasks was a mere mock condition, because it was always a task repetition that was excluded in N3, which was also not possible in the N4 condition. As the experimental design including the precues differs with respect to the designs usually used for investigating *n* – 2 repetition costs in task switching, it is possible that this effect in the baseline condition is an artifact of the experimental procedure. To rule out this possibility, we created a new experiment using the traditional cueing variant of the task switching procedure. Blocks with four tasks alternated with blocks in which one of the tasks was excluded. We assumed a reduction or reversal of *n* – 2 repetition costs in blocks with four tasks, as it was the case in Experiment 2.

## Experiment 3

### Method

#### Participants

Originally, 24 subjects participated. Three of them had to be discarded due to not following the instructions or not completing the experiment. The final sample consisted of 21 subjects (four male) with a mean age of 25.5 years (range: 20–34 years). For replicating the interaction of task sequence and number of tasks in trial *n* that was found in Experiment 2 with an effect size of ${\upeta}_{\textrm{p}}^{{}^2}$ = .31, this sample yielded a power of .81. All participants had normal or corrected-to-normal vision.

#### Stimuli, tasks, and apparatus

These were identical to Experiment 2 except for the following differences. No precues were presented in this experiment. Blocks with four tasks and blocks with three tasks alternated, the number of tasks in the first block being counterbalanced across participants.

#### Design and procedure

This was identical to Experiment 2, except for the following differences.

The experiment was run in a single session that took about 30 minutes. The test session consisted of six experimental blocks of 64 trials each.

Each trial began with the presentation of a fixation mark for 300 ms. Then, the task cue was presented for 600 ms. After that, the cue disappeared and the imperative stimulus was presented for 2,500 ms or until the participant’s response.

### Results

The practice block was not analyzed. Furthermore, the first three trials of each block were excluded, as were sequences involving an error in trials *n* – 2 or *n* – 1. From RT data, errors in the current trial were also excluded. A mean number of 75 trials per cell (*SD* = 12) was left for analyses. Mean individual LISAS were subjected to a repeated-measures ANOVA, with the within-subjects factors task sequence (CBA vs. ABA), and NTasks (N4 vs. N3).

**Fig. 6:**
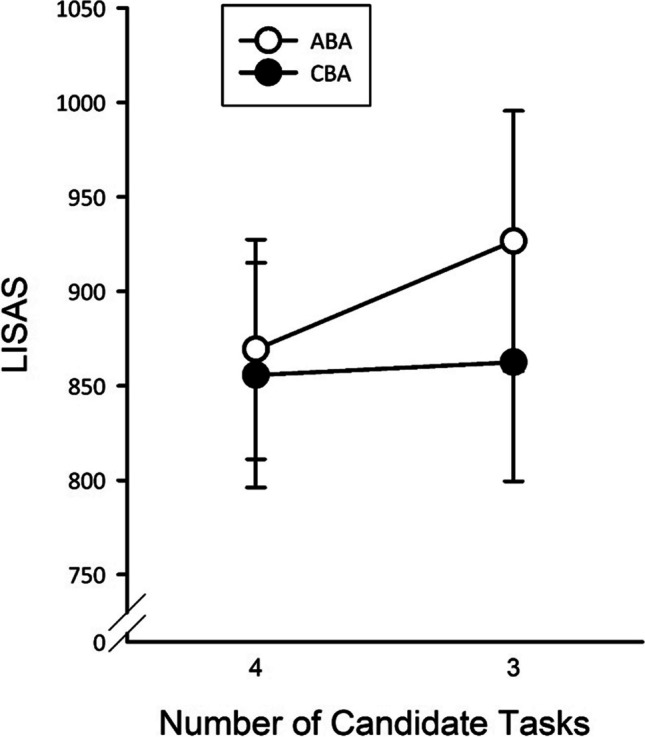
Experiment 3: Mean LISAS as a function of task sequence and number of candidate tasks in trial *n*. Error bars represent *SEM*

The main effect of task sequence was significant, *F*(1, 20) = 10.20, *p* < .01, ${\upeta}_{\textrm{p}}^{{}^2}$ = .34, due to higher LISAS for ABA sequences (898) than for CBA sequences (859). The main effect of NTasks was marginally significant, *F*(1, 20) = 3.38, *p* = .08, showing a trend towards higher LISAS with four (895) than with three tasks (862). Importantly, both factors interacted, *F*(1, 20) = 5.02, *p* < .05, ${\upeta}_{\textrm{p}}^{{}^2}$ = .20. *N* – 2 repetition costs were present in blocks with three tasks (*p* < .001) but were nonsignificant in blocks with four tasks (*p* = .43; cf. Fig. 6). Bayes factor paired *t* tests yielded an estimate of BF_10_ = 20.02 for blocks with three tasks and BF_10_ = 0.33 for blocks with four tasks, confirming the results of the ANOVA that *n* – 2 repetition costs were only present with three tasks.

### Discussion

Using the usual cueing variant of the task switching paradigm without the additional presentation of precues, the results of Experiment 3 replicate the finding of Experiment 2: Significant *n* – 2 repetition costs were found when participants switched among three tasks. In contrast, no *n* – 2 repetition costs were present when participants had to switch among four tasks. Therefore, it seems unlikely that the *n* – 2 repetition benefit with four tasks that was found in Experiment 2 was due to artifacts of the experimental procedure.

## General discussion

The present study aimed at investigating effects of a short-term reduction of the number of candidate tasks on *n* – 2 repetition costs. For this purpose, a variant of the task switching paradigm was used in which in some trials, a precue excluded a subset of all tasks as the relevant one in the upcoming trial. In Experiment 1, the number of candidate tasks was reduced from three to two in half of the trials. While significant *n* – 2 repetition costs were present in sequences without the information of this precue, they were reduced when one task could be excluded based on the precue in at least one of the three trials of the sequence. In Experiment 2, the number of candidate tasks was reduced from four to three and from four to two in one third of all trials, respectively. Significant *n* – 2 repetition costs were present in trials with three candidate tasks only.

First, the main effect of the precue (In Exp. 1) as well as its effect on *n* – 2 repetition costs suggests that participants actively used the information provided by the precue. This replicates the results of Kleinsorge and Scheil (2015, 2017), as does the reduction of switch costs by informative precues that was visible in the supplementary analysis of Experiment 1. This was the case even though the precue does not allow for task-specific preparation that may directly facilitate performance in the upcoming trial. Instead, it seems that the beneficial effect of the precue is due to a change in the way interrelations among individual tasks are represented. Kleinsorge and Scheil (2015) argued that switching among more than two tasks differs from switching among two tasks by requiring a more complex process of task selection because with more than two tasks, more complex constraints are implemented among the individual task representations. In contrast, selection among only two tasks may proceed by a simpler task selection mechanism that may exploit any single features the two tasks differ on. A number of three tasks constitutes the standard case in paradigms aiming at measuring *n* – 2 repetition costs. Thus, the present findings suggest an intimate link between the implementation of relatively complex task set representations and the representational basis of task set inhibition as underlying *n* – 2 repetition costs.

The question when and how inhibition in task switching might be released is still a matter of debate. The same holds for possible targets of inhibition. While some results support the notion of response-related processes underlying *n* – 2 repetition costs (e.g., Schuch & Koch, 2003), others provide evidence for task-specific activation as the target of inhibition (e.g., Gade & Koch, 2014; Scheil, 2016). Importantly, the present manipulation of the size of *n* – 2 repetition costs by a short-term reduction of the number of candidate tasks cannot be directly related to either response-related or preparation-based processes, as the relevant task is not directly indicated by the precue. Instead, only the task environment is changed on a trial-wise basis. A possible link between the establishment of antagonistic constraints and inhibition underlying *n* – 2 repetition costs might be the amount of conflict due to interference. It can be assumed that in the present paradigm, in which nontransparent cues, multivalent stimuli and overlapping response sets were used, between-task conflict is high. The possibility to create simpler task set representations in a given trial due to the information provided by the precue might therefore reduce conflict and, in turn, reduce the need for inhibition. Related to the computational model of Sexton and Cooper (2017), the exclusion of one of the tasks by the precue might reduce activation of the respective task demand unit. As a consequence, interference from that task and, therefore, between-task conflict is reduced, which should in turn reduce the amount of inhibition released by conflict monitoring units. In the present case, the residual amount of inhibition still necessary to deal with task conflict between the remaining two tasks may, on a behavioral level, have led to insignificant *n* – 2 repetition costs instead of an *n* – 2 repetition benefit that would be expected if no inhibition was released at all. Importantly, the present findings suggest that the interrelations among task demand units are not fixed but can be modified (by an establishment of simple antagonistic constraints) in a transient manner.

Kleinsorge and Scheil (2015) argued that the possibility to establish antagonistic constraints that may distinguish between two tasks based on a single feature is a unique characteristic of two-task environments. This was further corroborated by the fact that the reduction of switch costs due to the precue, which was observed for reductions from four to two tasks in Kleinsorge and Scheil (2015) and from three to two tasks in the present experiment, does not occur for a reduction from five to three tasks (van’t Wout et al., 2015). With reference to the model of Sexton and Cooper (2017), and assuming that a short-term reduction of the number of candidate tasks also reduced the activity of the conflict monitoring units associated with the excluded tasks, this suggests that beneficial effects on switch costs are only visible if the number of active conflict monitoring units is reduced to 1, whereas no reduction occurs if at least three conflict monitoring units are active. As a consequence, one may argue that the establishment of antagonistic constraints can be considered as a special variant of the Sexton and Cooper model in which only one conflict monitoring unit is active. However, one may also question whether the model of Sexton and Cooper (2017) offers a plausible account for larger sets of tasks: If conflict monitoring units are only able to handle conflict among two task demand units, their number should rise dramatically with larger sets of tasks according to the formula Number of Conflict Monitoring Units = (*n*² - *n*)/2, with *n* being the number of task demand units. Thus, with five task demand units, 10 conflict monitoring units would be needed, which rises to 15 with the addition of another task and to 26 with seven task demand units. Thus, it seems plausible to assume that larger numbers of tasks are represented in a more elaborated manner that exploits semantic interrelations or some other form of elaborate coding (cf. Kleinsorge & Apitzsch, 2012).

*N* – 2 repetition costs are usually observed when participants switch among (at least) three tasks. Investigating *n* – 2 repetition costs this way has hitherto been considered as a methodological requirement in order to be able to compare sequences of the forms ABA and CBA. Our present findings suggest that the tie of *n* – 2 repetition costs to three-task situations may not only be a methodological peculiarity but relate to the very origin of these costs. That is, when switching among only two tasks, the processes underlying *n* – 2 repetition costs may simply be reduced to a degree too low for this effect to occur. Furthermore, the above considerations suggest that with larger numbers of tasks, still other processes might come into play.

Related to the aforementioned point, a surprising finding was the lack of *n* – 2 repetition costs in trials with four tasks. This was not only observed with the special precue paradigm we applied in Experiments 1 and 2, but also in Experiment 3, where we used the standard task switching procedure. *N* – 2 repetition costs were present in blocks with three tasks but absent in blocks with four tasks. Generally, task switching studies using four tasks are not uncommon. In fact, this was even true for the studies from our lab that build the basis for the present experiments (Kleinsorge & Scheil, 2015, 2017). However, these studies usually focus on other effects than *n* – 2 repetition costs. For example, Katzir et al. (2018) used four tasks to examine the specificity of suppression of stimulus–response rules, but they did not include *n* – 2 repetition costs in their analyses. As studies investigating *n* – 2 repetition costs usually use three tasks for their experiments, the number of previous studies using more than three tasks to investigate this very effect is rather limited. To our knowledge, only Schuch and Grange (2015, Exp. 2) used four tasks. In their experiment, *n* – 2 repetition costs were present. However, tasks and procedure different quite substantially from our experiments, so it is not possible to draw firm conclusions here. In the present study, we expected to find *n* – 2 repetition costs in the condition with four tasks, because a task environment with four tasks should be even more complex than a condition with only three tasks, calling for a high amount of inhibition to reduce interference. The absence of costs cannot be explained with the idea of antagonistic constraints, because this concept does not differentiate between conditions with three and with four tasks. Related to the computational model of Sexton and Cooper (2017), the number of conflict monitoring units rises from three to six when the number of tasks increases from three to four. As a consequence, more units are actively spreading inhibition to increase the relative activation of the task demand unit belonging to the currently relevant task. Importantly, the lack of *n* – 2 repetition costs in the condition with four tasks can be explained by the Sexton and Cooper model if one additionally assumes that the total amount of inhibition that can be released by the conflict monitoring units is limited. In this case, the amount of inhibition per unit decreases as the total number of units increases, thereby minimizing *n* – 2 repetition costs. However, more research is needed to investigate this assumption and, more generally, the presence or absence of *n* – 2 repetition costs in conditions with more than three tasks.

An effect that may somehow be related to the present results are fadeout costs (Mayr & Liebscher, 2001; Pereg & Meiran, 2018). They can be observed when one of the two task sets is eliminated at some timepoint during a block of trials. Although this procedure indicates only one task set as being relevant for the rest of the block (thereby creating in fact a single task condition in which no task set selection is necessary), performance does only benefit after an initial adaptation phase (10 trials in the experiment of Mayr & Liebscher, 2001; one trial in the experiments of Pereg & Meiran, 2018). This delay is assumed to be due to a shift within the task set architecture from a task switching to a single task context that takes some time to be completed. The fadeout paradigm differs from the experimental approach used in the present study. Most importantly, the present short-term reduction does never reduce the set of candidate tasks to only one task. As a consequence, and in contrast to the fadeout paradigm, task set selection is always necessary. However, both experimental designs point towards a similar conclusion: Participants are able to adapt their behavior during the course of the experiment, based on instructions rather than on experience. This adaptation does not only occur for parts of the task set, but also for the way participants structure and reconfigure the whole task set architecture. This may bear implications for theoretical assumptions of how humans flexibly adapt their behavior to changes in the environment.

In conclusion, the present study investigated how a short-term reduction of the number of candidate tasks affects *n* – 2 repetition costs. Significant *n* – 2 repetition costs were present with three tasks but were absent with two candidate tasks. This result is interpreted in terms of antagonistic constraints that can easily be established when switching among only two tasks. In contrast, when switching among three tasks, complex task representations are needed that also require a higher amount of inhibition to reduce interference, causing *n* – 2 repetition costs. Future research should investigate whether and how *n* – 2 repetition costs can be observed in tasks environments with more than three tasks.

## Data Availability

Data will be made available on reasonable request. The experiments were not preregistered.
